# Cancer-Associated Membrane Protein as Targeted Therapy for Bladder Cancer

**DOI:** 10.3390/pharmaceutics14102218

**Published:** 2022-10-18

**Authors:** Adlina Roslan, Nurshahira Sulaiman, Khairul Asri Mohd Ghani, Armania Nurdin

**Affiliations:** 1 Laboratory of UPM-MAKNA Cancer Research (CANRES), Institute of Bioscience, Universiti Putra Malaysia, Serdang 43400, Selangor, Malaysia; 2 Department of Biomedical Sciences, Faculty of Medicine and Health Sciences, Universiti Putra Malaysia, Serdang 43400, Selangor, Malaysia; 3 Department of Urology, Faculty of Medicine and Health Sciences, Universiti Putra Malaysia, Serdang 43400, Selangor, Malaysia

**Keywords:** bladder cancer, membrane proteins, protein biomarker, targeted therapy

## Abstract

Bladder cancer (BC) recurrence is one of the primary clinical problems encountered by patients following chemotherapy. However, the mechanisms underlying their resistance to chemotherapy remain unclear. Alteration in the pattern of membrane proteins (MPs) is thought to be associated with this recurrence outcome, often leading to cell dysfunction. Since MPs are found throughout the cell membrane, they have become the focus of attention for cancer diagnosis and treatment. Identifying specific and sensitive biomarkers for BC, therefore, requires a major collaborative effort. This review describes studies on membrane proteins as potential biomarkers to facilitate personalised medicine. It aims to introduce and discuss the types and significant functions of membrane proteins as potential biomarkers for future medicine. Other types of biomarkers such as DNA-, RNA- or metabolite-based biomarkers are not included in this review, but the focus is mainly on cell membrane surface protein-based biomarkers.

## 1. Introduction

Bladder cancer (BC) is the most common malignancy of the genitourinary system, and the majority of patients are diagnosed between the ages of 50 to 70 years old [1,2]. It accounts for 3% of all current cancer cases treated worldwide, with 573,278 new cases and 212,536 deaths reported in 2020. Men have a threefold higher prevalence than women [3]; however, women are exposed to a higher risk of developing an advanced stage. The differences in incidence and outcomes between men and women are yet to be understood. Nevertheless, the disparity is thought to be due to exposure to environmental factors and differences in anatomical and physiological factors, including genetics and hormones [4].

BC is classified based on the depth of invasion of the lamina propria: (1) non-muscle invasive (NMIBC) and (2) muscle-invasive bladder cancer (MIBC). NMIBC is confined to the inner layer of the bladder wall (lamina propria) without invading the muscle layer, unlike MIBC, which can be found in the muscular wall of the bladder [5]. It is estimated that NMIBC is the most commonly diagnosed BC among cancer patients, representing about 75% of the population, while the rest of the patients suffer from MIBC and metastases. However, studies have shown that patients diagnosed with NMIBC have a high recurrence rate of up to 70% within five years [6,7], and up to 30% of patients die from the disease [8]. Even more alarming is that approximately 5 to 20% of patients will progress to invasive carcinoma [7]. Hence, constant efforts need to be performed to reduce the incidence of the disease, as the cases not only affect individuals but can also be an economic burden as the need to support the medication for patients increases.

Current treatments such as chemotherapy are commonly used to treat BC patients but upstaging of the present tumours still occurs [9]. Some arguments propose that the cause of tumour resistance to the chemotherapeutic agent used during therapy is due to the alteration of membrane proteins (MPs) on the surface of the cancer cells [10,11]. This alteration is discussed further in this review. Hence, MPs are of great interest to researchers to investigate their capability and sensitivity as targeted biomarkers for various cancers, including BC. Several types of biomarkers are currently used in cancer diagnosis, including DNA-, RNA-, and metabolite-based biomarkers [12], but this review focuses only on protein-based biomarkers, particularly MPs. The review presents studies on MPs as potential biomarkers, including their types and significant functions that are valuable in the treatment of BC. The use of proteomics platforms for biomarker discovery and the roles of MPs are also discussed.

## 2. Materials and Methods

In this review, we performed a comprehensive search using search engines such as ‘PUBMED’, ‘Science Direct’, and ‘Google Scholar’ to select relevant articles using the following keywords: ‘bladder cancer’, ‘biomarkers’, ‘membrane proteins’, ‘targeted therapy’, and ‘diagnosis’. Conference abstracts and articles that were not written in English were excluded. Of the 257 articles found, 124 relevant articles were considered in this review.

## 3. Stages of Bladder Cancer

NMIBC can be divided into (i) flat, poorly differentiated tumours confined to the mucosa (Tis), (ii) tumours invading the epithelium or mucosa (Ta), and (iii) tumours invading the subepithelial connective tissue or laminar propria (T1). As summarized by Magers and colleagues [13], bladder carcinoma can progress starting from the Tis, Ta, and T1. It can become invasive into the inner half of the muscular layer (T2a) and the outer half of the muscular layer (T2b). Cancer may then progress to the microscopic or macroscopic invasion of the fatty tissue (MIBC) (T3a and T3b). At stage T4, the cancer cells invade adjacent or neighbouring organs, such as the prostate in men.

## 4. Current Status of Biomarker in Bladder Cancer

The most popular non-invasive technique for detecting and monitoring BC is urine cytology. Although this method has roughly 80–85% specificity, its applicability is limited by poor sensitivity (40–50%) [14]. Few protein-based diagnostic biomarkers have been established and approved by the Food and Drug Administration (FDA) for BC detection and preventing tedious cystoscopies. These include the nuclear matrix proteins (NMPs) and bladder tumour antigens (BTA) [15]. To date, no MP has been established as a biomarker in BC and has not yet entered the development phase. Several have been found in the early stage (phase 1 or 2) with a small sample of clinical trials, but there is currently a lack of extensive clinical trials. For this reason, there is little data on MPs as biomarkers in BC, and only a few details can be reported on the status of clinical application. More collective efforts should be carried out to discover the potential MPs and studies on their involvement in recurrence, the prognosis of clinical outcome or prediction, and the efficacy of chemotherapy.

## 5. Targeted Therapy for Bladder Cancer

A combination of cisplatin-based chemotherapy is a standard method of reducing recurrences in BC. However, conventional chemotherapy leads to cell resistance to the drugs used during therapy [16]. Following this, targeted therapy has emerged as the accepted treatment for BC patients. In targeted therapy, a drug targets the oncogenic drivers on the tumour cells [17] to reduce the undesirable effects. Hence, it is crucial to identify the targets to develop targeted cancer therapies. In a study by Hanahan and Weinberg, the six hallmarks of cancer were proposed to better understand how to identify the targets [18]. Table 1 summarises the six hallmarks of cancer and how they relate to the membrane-associated proteins involved in the process. Although only several of the MPs were examined in the study, the presence of the MPs can be seen to be impactful and plays an essential role in the progression of cancer cell cycles, particularly as upstream regulators of activity in the cell. By understanding the hallmarks of cancer, various targets for targeted therapies can be proposed, including MPs.

## 6. Membrane Proteins: Structural and Biological Role in Cancer

**Figure 1:**
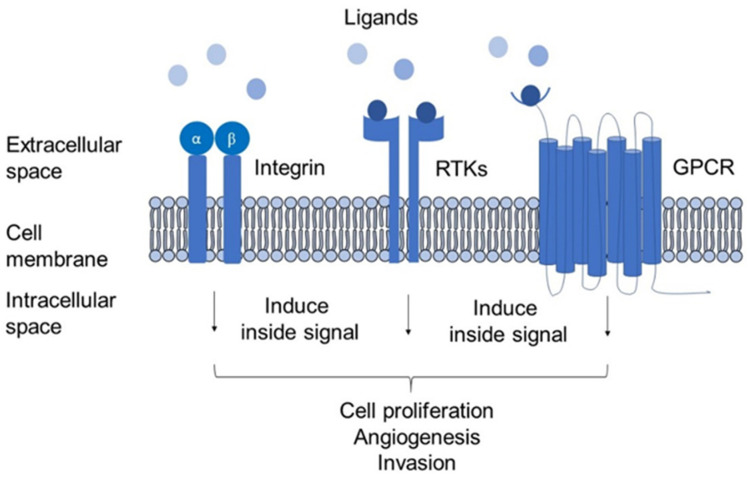
Membrane proteins induce cell responses leading to cancer.

MPs are proteins found in cell membranes, including receptors or transporters, and account for approximately 80% of drug targets [19]. It is important to note that various modified MPs expressed by cancer cells can trigger downstream signalling that leads to cancer behaviour. Common types of membrane protein receptors include integrins, receptor tyrosine kinases (RTKs), and G-protein coupled receptors (Figure 1). Previous studies have found that these proteins are responsible for altering the normal cell cycle, which results in proliferation, cell survival, apoptosis, invasion, angiogenesis, and other abnormal processes. The accessible structure of membrane proteins on the cell surface becomes one of the most valuable structural characteristics to be a drug target and an ideal biomarker. Moreover, MPs are the first to be altered by changes that occur in the cellular microenvironment as they are located on the cell surface. MPs can be shed from the cell surface into biological fluids such as urine as part of the signalling pathway to a pathogenic insult [20]. The number of MPs in these fluids can offer crucial diagnostic and prognostic data on disease prevalence, severity, and efficacy.

### 6.1. Integrins

The integral membrane glycoproteins, also called integrins, are one of the cell membrane receptors. They are commonly known to provide connections, respond to the extracellular matrix (ECM), including collagen and elastin [21], and mediate cell response. The integrin structure consists of α and β subunits of transmembrane glycoproteins that are not covalently linked. The dimerisation of the integrin subunits is essential to facilitate cell migration, adhesion, and proliferation. The structure of the active form of integrin is crucial as it triggers multidirectional signalling of downstream signals and cell responses. Altered integrins play a role in cancer progression, including initiating cancer cell proliferation and cell survival [22]. Upregulation of integrin has been reported to increase adherence to E-cadherin, which is thought to prevent the dissociation of cancer cells leading to metastasis. For instance, overexpression of αEβ7 integrin, integrin α2β1, and integrin β8 has been reported to act as a driving force in the progression of BC and the development of resistance to therapy [23,24]. Thus, integrins may serve as one of the potential biomarkers.

### 6.2. Receptor Tyrosine Kinases (RTKs)

Another transmembrane protein is the receptor tyrosine kinases (RTKs), which have a single transmembrane domain. RTKs control vital biological functions such as proliferation, apoptosis, differentiation, and metabolism. RTKs are frequently overexpressed in BC, resulting in downstream signalling pathways [25]. Previous publications indicate that multiple RTKs families have been found in various cancers, for instance, VEGFR-2 in BC [26] and TACSTD2 in breast cancer [27], non-small-cell lung cancer [28], and thyroid cancer [29]. The studies also suggest that the RTKs family is an important marker for cancer progression. Numerous protein tyrosine kinases are found to be overexpressed and/or oncogenically altered in human malignancies, making targeted therapy against protein tyrosine kinases challenging.

### 6.3. G-Protein Coupled Receptor-Chemokine Receptor

The G-protein coupled receptors (GPCRs) are also among the membrane receptors that comprise many genes—an estimated 800 genes in total in the human genome [30]. These proteins also have an extracellular region (the N-terminus) for the accessibility of ligands for binding and domains for recognition. Intracellular signalling can be triggered upon conformational changes in the receptor protein that lead to altered binding of GTP-binding proteins and activation. The proteins thus play a role in the signalling pathways by binding to the extracellular region [21]. The chemokine receptor is one example of a membrane protein with a structure of seven transmembrane proteins and is known to belong to the GPCR family [31]. This receptor, which is expressed by cancer cells, plays a role in cancer progression by promoting metastasis, cell survival, and proliferation. It has also been used as a molecular marker for targeted therapies, such as chemokine receptor 7 (CXCR7) in BC [32], CXCR2 in breast cancer [33], and CXCR5 in prostate cancer [34]. Furthermore, Takanami et al. [35] revealed that chemokine receptor 7 (CCR7) expression is associated with cell migration to lymph nodes and subsequent lymph node metastasis. Hence, the chemokine receptor is also a possible relevant membrane protein that could be an ideal biomarker in various cancers, and further studies on its significance in BC need to be performed.

## 7. Mechanism of Bladder Cancer Recurrence

Treatments for BC, such as chemotherapy, are considered effective, but responses to the tumour are short-lived, and recurrence is common. The cancer recurrence after chemotherapy could be due to the resistance mechanism of the cancer cell to various anticancer drugs, which interfere with cancer treatment. The resistance to anticancer drugs is associated with the overexpression of some MPs [10]. By altering their membrane composition, lowering drug transporters, and enhancing efflux pumps, tumour cells prevent the accumulation of chemotherapeutic drugs [36]. Chemotherapy resistance is also influenced by the change or loss of the drug target. The cancer recurrence mechanisms are further discussed below.

### 7.1. Modification of Drug Efflux

Abnormalities in cell death mechanisms are associated with the chemotherapy resistance that develops in most cancers. Tumour cells employ several strategies to limit drug penetration, such as lowering drug uptake or enhancing drug efflux. Diffusion, active transport, and endocytosis are the three main routes by which drugs enter tumour cells [37]. Immunotoxins that enter tumour cells by endocytosis are ineffective against cancer cell mutants with dysfunctional endocytosis [36]. For instance, the expression of P-glycoprotein-1 (Pgp-1), multidrug resistance proteins (MRPs), that act as drug efflux pumps, has been linked to chemotherapy resistance [37,38]. In numerous cancer cell lines, modification of their activities is linked to chemosensitivity to doxorubicin in vitro [39,40]. These include MRP1, MRP2, and MRP3, which are highly expressed in BC after chemotherapeutic treatment. Modulation of these transporters (MPs) reduced doxorubicin uptake, resulting in resistance to the drug [10].

### 7.2. Induction of Drug-Detoxifying Mechanism

On the other hand, the induction of a drug-detoxifying mechanism also contributes to chemotherapy resistance. Inhibition of drugs and lack of physiological stimulation are both exclusive to certain drug classes [41]. For example, platinum drugs, including cisplatin, and carboplatin, can be inactivated by specific binding to the thiol glutathione. This reduces the ability of native drugs to reach their target [39,41] and causes drug efflux via ABC transporter proteins 9 (MPs) [42]. Thus, overexpression of Pgp-1 and MRP1 (ABC transporters) in human BC could be considered a plausible component of the multifactorial mechanisms of drug resistance [10].

### 7.3. Modification of Drug Targets

Alteration of the drug target, including mutations or modifications in expression levels, also affects sensitivity to the drug [39,41]. For example, numerous protein tyrosine kinases, have been found to exhibit overexpression and/or oncogenic alterations in human malignancies, making targeted therapy against protein tyrosine kinases challenging. Indeed, the mutation in non-small-cell lung tumours leads to a dramatic reduction in the efficacy of epidermal growth factor receptor (EGFR) inhibitors [41]. Overexpression of the protein tyrosine kinase EGFR (MPs) has also been found in BC [43]. Amplification and mutations at this EGFR could therefore lead to drug resistance [36].

### 7.4. DNA-Damage Repair

Most chemotherapeutic drugs cause DNA damage in tumour cells, either directly or indirectly, in the case of platinum-based therapies and topoisomerase inhibitors [42]. In resistant cancer cell lines, DNA topoisomerase-II, a target of doxorubicin, has been altered [42]. A reduction in DNA damage is observed when the drug target is reduced by post-transcriptional modifications, including ubiquitination [44,45]. This leads to DNA-damage repair. The DNA-damage response factors would rapidly detect the damage in normal cells and lead to the activation of cell cycle checkpoints and DNA repair [46].

### 7.5. Activation of Prosurvival Signalling Pathways

On the other hand, cancer cells establish novel prosurvival signalling pathways in response to chemotherapy. The tumour cells generate pathways for survival or defects in programmed cell death to prevent apoptosis, leading to drug resistance. In individuals with EGFR-mutant lung malignancies, a deletion in the Bcl-2 interacting mediator of the cell death gene is substantially associated with resistance to protein tyrosine kinase inhibitors [41]. A variety of proteins are involved in these signalling pathways, including oncogenes (such as *RAS*), tumour suppressor genes (such as *TP53*), and survival proteins (such as nuclear factor-kB and transcription activator 3) [39,41,47]. Higher EGFR expression (MPs) leads to excessive cell proliferation. It also causes an increase in angiogenesis and a decrease in apoptosis, both of which are required for further malignant growth [43]. Mutations, amplifications, chromosomal translocations, and overexpression of these genes have therefore been linked to resistance to chemotherapy and targeted therapies in various cancers [48].

## 8. Membrane Proteins as Targeted Therapy for Cancer

Discovering biomarkers helps in the diagnosis of cancers, including BC [49], and it is becoming increasingly crucial as cases of BC recurrence increase. Researchers are constantly striving to identify potential biomarkers to diagnose cancer at an early stage. Although the use of MPs as biomarkers in BC has been introduced in recent years, there is still a lack of established MPs that are more specific and sensitive. Table 2 summarises some of the proposed MP biomarkers in BC, while Table 3 summarises several studies on their expression in other cancers. Some studies utilised proteomic-based approaches for cancer detection, such as immunohistochemistry (IHC), but some also used gene expression profiling, such as RT-PCR. The proposed MPs such as P-glycoprotein-1 (Pgp-1), Her2/Erb-b2 receptor tyrosine kinase 2, tumour-associated calcium-signal transducer 2 protein (TACSTD2), vascular endothelial growth factor receptor 1 (VEGFR1), and others were not only valuable for the diagnosis of BC but could also facilitate and improve clinical outcomes in multiple cancer types. The determination of MPs could pave the way for personalised medicine for cancer patients by developing a wide range of diagnostic biomarker assays that correspond to variations in individual patients [50]. This suggests that further studies should be conducted to identify other MPs that are potentially responsible for disease progression in BC.

### 8.1. Pgp-1

P-glycoprotein, also known as multidrug resistance 1 (MDR1), is a membrane-associated protein that belongs to the superfamily of ATP-binding cassette (ABC) transporters. Various substances are transported by ABC proteins through extracellular and intracellular membranes. The seven different subfamilies of the ABC genes are *ABC1*, *MDR/TAP*, *MRP*, *ALD*, *OABP*, *GCN20*, and *White*. The MDR/TAP subfamily contains this protein as a member. Multidrug resistance is caused by members of the MDR/TAP subfamily. This protein functions as an ATP-dependent drug efflux pump for xenobiotic substances with a wide range of substrate specificity. It controls and reduces drug uptake into cells and frequently prevents the development of anticancer drug resistance. The blood-brain barrier also uses this protein as a transporter [51]. An analysis of surgically removed clinical specimens from 47 bladder tumours reported a significant association between the expression of Pgp-1 and response to doxorubicin. Although the resistance mechanism remains unclear, it has been suggested that drug sensitivity to doxorubicin may decrease in BC cancer patients due to higher expression of this protein [10,52].

### 8.2. Her2

Her2 or also known as Erb-b2 receptor tyrosine kinase 2, is a component of numerous cell surface receptor complexes. It forms a heterodimer with other members of the EGFR family when the ligand is present. This stabilises the ligand binding and enhances kinase-mediated activation of downstream signalling cascades. It also controls peripheral microtubule stabilisation and growth. Amplification of this gene and/or overexpression of the protein has been found in numerous malignancies, such as breast, ovarian, and bladder tumours [51,53]. In contrast to breast cancer, where the efficacy of Her2-targeting drugs in metastatic and adjuvant situations has been well-established for a decade, comprehensive clinical trials of BC are currently lacking. In certain small-sample studies, Her2 receptor-targeting therapy has demonstrated a favourable clinical outcome, with up to 70% of the overall response rate (phase two) reported in patients with advanced NMIBC treated with trastuzumab, carboplatin, gemcitabine, and paclitaxel. However, further phase three and four clinical trials are required before the involvement of this receptor in BC can be definitively established [54,55].

### 8.3. TACSTD2

Receptor tyrosine kinase is a calcium signalling receptor found on cell surfaces. It is known to be a cancer-related antigen and potentially serves as a growth factor receptor. Gelatinous drop-like corneal degeneration and numerous cancers, including BC, have been linked with mutations in this gene [51,56]. In a clinical trial (phases one and two) with sacituzumab govitecan (an antibody-drug conjugate that targets TACSTD2 in metastatic BC), an overall response rate of 27% was reported among BC patients. Overexpression of this protein could serve as a potential MP biomarker in BC, but the signalling pathways and regulatory factors of its expression are still unclear [57,58].

### 8.4. VEFGR1/2

VEFGR1/2 is a member of the VEGFR family and receptor tyrosine kinases (RTKs) that have a transmembrane segment, a tyrosine kinase (TK) domain within the cytoplasmic domain, and an external ligand-binding region with seven immunoglobulin (Ig)-like domains. MPs play a crucial role in regulating angiogenesis, the development of embryonic vasculature, cell survival, migration, macrophage function, chemotaxis, and tumour cell invasion. It is crucial for angiogenesis and vasculogenesis as it binds to VEGFR-A, VEGFR-B, and the placental growth factors. Vascular endothelial cells, placental trophoblast cells, and peripheral blood monocytes express this receptor. VEGFR-1 and VEGFR-2 have both common and unique ligands [51]. In a phase two trial, a combination of bevacizumab with gemcitabine-carboplatin exhibited a response rate of approximately 50% in patients with advanced BC who had never received chemotherapy and were not eligible for cisplatin treatment [59].

### 8.5. Integrin β8

This protein forms a heterodimeric integrin complex by binding non-covalently to an alpha subunit. Integrin complexes typically mediate cell–cell and cell–extracellular matrix interactions and this complex contributes to the proliferation of human airway epithelial cells. It is a fibronectin-1 receptor that identifies its ligands by the sequences R-G-D. Integrin alpha-V: beta-6 promotes the R-G-D-dependent release of transforming growth factor beta-1 (TGF-beta-1) from regulatory latency-associated peptide and, therefore, plays a crucial role in TGF-beta-1 activation on the surface of activated regulatory T-cells (investigational). It is also associated with vascular formation, and the gene of this protein is known to be a cancer-related gene [51]. A study reported that the protein may be responsible for promoting and enhancing resistance to mitomycin C and hydroxycamptothecin, as well as cell proliferation. Liu et al. [24] proposed that stimulation of phosphorylated Y-box binding protein 1 mediates activation of prosurvival signal (c-Myc) and anti-apoptosis pathway (nuclear factor-κβ (NF- κβ) and β-cell lymphoma 2 (BCL2) signals), leading to multidrug resistance. The BC cells were successfully prevented from continuously growing and developing treatment resistance by targeting the integrin β8 with the Arg-Gly-Asp-pharmacological ser. Following this research, this integrin was discovered as a potential diagnostic and prognostic marker for BC, offering a novel therapeutic strategy [24].

### 8.6. FGFR3

FGFR3A is a protein that belongs to the FGFR family, whose amino acid sequence is highly conserved both within and between different species. Members of the FGFR family vary from one another in terms of their tissue distribution and ligand affinity. Three immunoglobulin-like domains, a hydrophobic membrane-spanning segment, and a cytoplasmic tyrosine kinase domain form the extracellular portion of a full-length representative protein. The protein’s extracellular region interacts with fibroblast growth factors to trigger downstream signals that ultimately impact mitogenesis and differentiation [51]. The mechanisms of resistance to FGFR inhibitors have not yet been reported in clinical studies; however, the study suggested that amplifications or mutations in proteins associated with EGFR, MET, RAS, and PI3K signalling are the mechanisms that cause activation of bypass signalling. Several clinical trials (phases one and two) are now ongoing, with up to 40% overall response rate [60,61]. Further studies should be conducted to demonstrate the therapeutic efficacy of this protein and to evaluate the potential value of FGFR modifications as biomarkers.

### 8.7. CXCR7

CXCR7A is a chemokine receptor that regulates chemokine concentration and distribution through high-affinity chemokine binding dissociated from conventional ligand-driven signal transduction pathways, which leads to chemokine sequestration, degradation, or transcytosis. It is commonly classified as a chemokine-scavenging receptor, interceptor (internalising receptor), or chemokine decoy receptor. It serves as a receptor for the chemokines SDF1 and CXCL11. The binding of chemokines leads to the recruitment of beta-arrestin, resulting in ligand internalisation and activation of the MAPK signalling cascade. It is not only involved in cell adhesion but also promotes cell growth and survival of cells. The gene is known to be cancer-related [51]. A study reported that EGFR and Akt-signalling are plausible mechanisms by which CXCR7 stimulates BC cell proliferation and motility. High-grade cancer and metastasis are associated with higher CXCR7 expression, found in BC tissues and exfoliated cells [62]. As there are no comprehensive clinical studies on this protein, further studies should be conducted to better understand the role of this protein and the association of its expression in BC.

**Table 2:** Proposed membrane proteins for diagnosis and prognosis of bladder cancer.

No.	Protein Biomarkers	Types of Proteins/Receptors	In Vitro		Clinical Study	
			Method of Detection	Expression in Cell Lines	Method of Detection	Expression in Specimens
1.	Pgp-1	Transporter	WB	Highly expressed in 253J and J82 cells [63].	IHC	Highly expressed in 39 of 55 BC specimens (71%) (China) [63].
2.	Her2	RTK	WB	Expressed in BC cell lines but 10-fold lower in the breast cancer cell, SKBR3 cells [64].	IHC	Overexpressed more in NMIBC patients (21%) (China) [65]
3.	TCSTD2	RTK	RT-PCR	Highly expressed in multiple BC cell lines [50].	IHC	Highly expressed in 27.3% of the 99 patients (Japan) [66].
4.	VEGFR1	RTK	Immunoblot	Higher expression in TCCSUP [67].	IHC	Increased 2-fold in BC specimens compared with the normal (USA) [67].
5.	VEGFR2	RTK	Immunoblot	Highly expressed in J82 and HT1376 BC cells [67].	IHC	Increased 55% in BC specimens compared with the normal (USA) [67].
6.	Integrin β8	Integrin	Immunofluorescence assay	Overexpressed in Biu87 and T24 BC cells [24].	IHC	Increased 2-fold higher in highly malignant BC (China) [24].
7.	FGFR3	RTK	WB	Highly expressed in RT4, RT112, and SW780 cells [68].	RT-qPCR	Highly expressed (40.0%) in patients with pT1 BC (Korea) [69].
8.	CXCR7	GPCR	Q-PCR	Highly expressed (3-10-fold) in 5637 and HT1197 cell lines than in other cell lines [62].	IHC	Highly expressed (5–10-fold) in BC tissues than in normal tissues (Florida, USA) [62].

Abbreviations: Pgp-1, P-glycoprotein-1; Her2, Human epidermal growth factor receptor 2; VEGFR1, Vascular endothelial growth factor receptor 1; VEGFR2, Vascular endothelial growth factor receptor 2; CXCR7, Chemokine receptor 7; FGFR3, Fibroblast growth factor receptor 3; IHC, immunohistochemistry; RT-PCR, reverse transcriptase-PCR; RT-qPCR, reverse transcriptase- quantitative PCR; WB, western blot.

**Table 3:** The expression of the proposed membrane protein biomarkers in other cancer based on in vitro and in vivo studies.

No.	Protein Biomarkers	Types of Proteins/Receptors	Chemotherapy Resistance	Expression in Other Cancer	In Vitro		Clinical Study	
					Method of Detection	Expression in Cell Lines	Method of Detection	Expression in Specimens
1.	Pgp-1	Transporter	Reported with paclitaxel and docetaxel resistance [70]. Increased chemotherapeutic drug outflow in cell culture, which promotes multidrug resistance [71].	NSCLC	Immunofluorescence assay	Expressed in SPCA1, lung cancer cell line and downregulated by Verapamil [72].	IHC	Expressed in 52/60 NSCLC patients treated with Docetaxel and 46/60 patients in the docetaxel + tamoxifen group (China) [73].
				Breast	WB and immunofluorescence	Highly expressed in MCF-7R and MCF-7 breast cancer cell lines [74].	IHC	Expressed in 9/49 patients in the pretreatment group and increased to 29/49 after chemotherapy (India) [75].
				Stomach (gastric)	WB	Expressed in SGC-790, gastric cancer cell line, increased when stimulated with Paclitaxel [76].	RT-PCR, IHC	Expressed in 54 gastric patients (61.3%) with low IRF-1 expression (China) [77].
2.	Her2	RTK	Reported with cisplatin-based regimens resistance [78].	Ovarian	WB	Overexpressed in Caov-3 ovarian cancer cells.	IHC	Highly expressed (79%) in samples of recurrent ovarian cancer (Taiwan) [79].
3.	TACSTD2	RTK	Reported with tamoxifen and trastuzumab resistance [57,80].	NSCLC	WB	Upregulated in human NSCLC, A549, NCI-H520, NCIH441, and NCI-H226 cell lines, compared to normal HBE cell lines [28].	RT-PCR, IHC	1.5-fold up-regulated in 58/107 NSCLC patients and increased in the advanced cancer stage (China) [28].
				Thyroid	WB	Overexpressed and promoted the invasion and migration of K1, FTC-133, and 8505C thyroid cancer cell lines [29].	RT-PCR, IHC	Highly expressed (53.1%) in malignant thyroid tissues of 51/96 patients (China) [29].
				Breast	WB	Expressed higher in the breast cancer cell line (MCF-7 and MDA-MB-231 cells) than normal breast cell line (MCF-10A) [81].	RT-PCR, IHC	Expressed with 1.55 ± 0.78 fold higher in 20 pairs of breast cancer tissues than in adjacent tissues (China) [81].
4.	VEGFR1	RTK	Reported with bortezomib resistance [82]. Alterations of VEGFR1 contributed to resistance to anti-VEGF therapy [83].	Ovarian and Cervical	RT-PCR	Expressed low in an ovarian carcinoma cell line (DOV13) [84].	IHC	Significantly expressed in 8 patients with post-radiotherapy relapsed/persistent cervical cancer (Japan) [85].
				Colon	WB	Express in human colon cancer (HCT116 cell line) [86].	RT-PCR	Expressed in 39 metastatic colorectal cancer (Italy) [87].
				NSCLC	RT-PCR and WB	Induced expression at higher concentrations in NSCLC cell lines (A549 and SKMES1), when treated with Trichostatin A (TSA), compared to untreated [88].	RT-PCR	Expressed in 23 (85.2%) of 27 malignant specimens (Greece) [89].
5.	VEGFR2	RTK	VEGF-C/VEGFR2 signalling promotes tumorigenicity and potentially contributes to bevacizumab resistance [90].	Ovarian	RT-PCR	Expressed in an ovarian carcinoma cell line (DOV13) but low in levels [84].	WB	Express in 70 (92.1%) of 76 ovarian cancer tissues (Europe) [91].
				NSCLC	RT-PCR and WB	Expression increased in NSCLC cell lines (A549 and SKMES1) when treated with Trichostatin A (TSA) compared to untreated [89].	RT-PCR	Expressed in 24 of 27 (88.9%) malignant specimens (Greece) [89].
				Colon	WB	Weakly expressed in human colon cancer (HCT116 cell line) [86].	RT-PCR	Expressed in 72 metastatic colorectal cancer patients (Italy) [87].
6.	Integrin β8	Integrin	Correlated with resistance to gefitinib [92].	Pancreatic	WB	Highly expressed in a pancreatic cancer cell line (Panc-1) [93].	IHC	3.1-fold upregulated in pancreatic cancer patients (78 patients) [93].
				Colon	WB	Weakly expressed in human colon cancer (HCT116 cell line) [86].	RT-PCR	Expressed in 72 metastatic colorectal cancer patients (Italy) [87].
7.	FGFR3	RTK	Overexpressed FGFR3-S isoform was reported to be resistant to docetaxel [94].	NSCLC	RT-PCR & WB	Increased in A549 and NCIH460 NSCLC cell lines with exposure to nicotine [95].	IHC	Highly expressed FGFR3 in 3.3% NSCLC tumour samples (Netherlands) [96].
8.	CXCR7	GPCR	Overexpression of CXCR7 constitutes a mechanism of resistance to EGFR tyrosine kinase inhibitors [97].	Bladder	RT-PCR & WB	Expressed higher in RT4 compared to J82 and T24 cells [32].	IHC	Expressed highly in high-grade 2 BC specimens (33 of 78) (CA) [32]
				Breast	Flow cytometry	Expressed in breast cancer cell lines (4T1) [98].	IHC	Highly expressed in 106 of 109 human breast cancer specimens (97%) (CA) [98].

Abbreviations: Pgp-1, P-glycoprotein-1; Her2, Human epidermal growth factor receptor 2; VEGFR1, Vascular endothelial growth factor receptor 1; VEGFR2, Vascular endothelial growth factor receptor 2; CXCR7, Chemokine receptor 7; EGFR, Epidermal growth factor receptor; FGFR3, Fibroblast growth factor receptor 3; NSCLC, Non-small-cell lung cancer; IHC, immunohistochemistry; RT-PCR, reverse transcriptase-PCR; WB, western blot; ND, not detected.

## 9. Proteomic Platforms for Biomarker Discovery

Discovering a biomarker requires several steps, including sample collection, processing, acquisition of the data, and subsequent data analysis [99]. Biomarker discovery would be challenging without a specific instrument to analyse the protein of interest. Table 4 shows proteomics platforms for biomarker discovery in BC, including global and targeted approaches. All platforms (two to seven) share the common principle of using antibodies or probes to detect the biomarker referred to as targeted, with the exception of LC-MS/MS, which uses the global analysis approach. The available platforms were discussed to provide a better insight into the process of MP biomarker discovery.

The most recent generation of mass spectrometers has advanced the field of proteomics to the point where robust data collection can be identified, quantified, and monitored. This opens up a world of possibilities for cancer research [99]. Discovery-based studies are still the most widely utilised approaches in MS-based proteomics, often referred to as shotgun proteomics. It can be separated into two types: (1) label-based technologies that use isotopic or isobaric tags and (2) label-free MS-based proteomics [100]. The proteins (tagged or unlabelled with stable isotopes) are enzymatically digested and injected into liquid chromatography. They are then ionised and detected in a mass spectrometer. The peptides were analysed based on the *m*/*z* ratio. Following the processes, the *m*/*z* of the ionised fragmented peptides is measured. This yields the sequences of the peptides of the original sample [101,102].

In label-based technologies, proteins are labelled with stable isotopes by metabolic integration into living systems. For instance, stable isotope labelling by amino acids in cell culture (SILAC) [103,104]. Cells are cultured in a light or heavy culture media with stable isotope-labelled amino acids. Protein quantification is conducted after MS analysis by comparing light/heavy peptide pairings. In addition, several in vitro chemical labelling approaches, such as isotope-coded affinity tags (ICAT) [105], cleavable isotope-coded affinity tags (cICAT) [106,107], dimethyl labelling [108,109], and isobaric labelling [110], have been developed over the last two decades. ICAT offers two types of biotin-containing thiol-reactive tags: a “light” version without deuterium atoms (1H) and a “heavy” version with eight deuterium atoms (2H). The labelled proteins are mixed and degraded to peptides after labelling. Subsequently, the cysteine-containing peptides are enriched by affinity chromatography and quantified by MS [105]. An example of an isobaric labelling method is the isobaric tags for relative and absolute quantification (iTRAQ), which have been developed over the last two decades [110,111].

In label-free techniques, samples are compared by scanning MS2 spectra or by evaluating the chromatographic peak area [112,113]. Because both systematic and non-systematic variables influence the MS results, label-free techniques are less reproducible and accurate than stable isotope labelling approaches [114]. Label-free methods, on the other hand, have some advantages. These include an unlimited number of samples, more effective identification, and quantification of proteins [115], and a greater quantitative dynamic range.

On the other hand, proteotypic peptides (PTPs), the specific amino acid sequences, need to be monitored to identify proteins in a targeted approach [116]. The PTPs serve as a unique identifier for the targeted protein, and they’re tracked during the experiment. Targeted proteomics requires the analysis of precursor and fragment ions of the previously selected peptides. Due to this, the identification of peptides that best reflect the target protein is a crucial step in sample analysis [101,117,118]. Since targeted techniques are motivated by a specific hypothesis, prior knowledge of the target protein is required. Because the data for selecting the candidate peptides can come from various sources, it may be necessary to rely on previous research discoveries or general screening based on proteomic assays to obtain trustworthy outcomes [119]. Prior to the experiment, all information on the target peptides, including the optimal criteria for separation, ionisation, and fragmentation, must be determined using the traditional selected reaction monitoring (SRM) approach. As mentioned above, a solid selection of PTPs and peptide fragments ensures high sensitivity and specificity; such a method requires access to a wealth of information on the target proteins.

**Table 4:** Proteomic platforms of biomarker discovery.

No.	Platforms	Principle	Advantages	Drawbacks	Utilisation in BC Studies
1.	Liquid chromatography-tandem mass spectrometry (LC-MS/MS)	Ionise, fragmented molecules, and analyse the ions produced based on the mass-to-charge ratio (*m*/*z*).	Specific, sensitive, no antibody required.	Complexity	[120]
2.	Enzyme-linked Immunosorbent assay (ELISA)	Immobilise antigen to a plate-based surface and interact with an enzyme-linked antibody.	Cost-effective and easy to use.	Require high-quality antibody, possible unspecific binding.	[121]
3.	Western blot	Proteins are separated based on molecular weight, transferred onto a membrane, and detected with antibodies.	Separate based on molecular weight.	Require a large amount of protein, high-quality antibody, and possible unspecific binding.	[81]
4.	Immunohistochemistry (IHC)	Antibodies bind specifically to proteins.	The localised protein of interest.	The semi-quantitative assay requires high-quality antibodies and possible unspecific binding.	[81]
5.	Surface-enhanced Raman spectroscopy (SERS)	Vibrational optical spectroscopic technique based on strong interactions between proteins and metal nanoparticles.	Little sample preparation.	Unspecific interaction, less sensitive.	[122]
6.	Colorimetric assay	Change of colour due to an enzymatic or chemical interaction between spotted reagents and the analyte.	Fast, inexpensive.	Stability/shelf-life, possible unspecific interaction	[123]
7.	Electrochemical ELISA-based assay/ biosensor	Requires specific markers to promote selective binding and detection. This biomolecular recognition takes place close to the functionalised surface of an electrode.	Versatile	Long optimisation, possible unspecific binding.	[121]

## 10. Conclusions

The discovery of new biomarkers that can be used in the diagnosis and treatment of cancer is crucial for the development of personalised medicine. Successful drug development can lead to an efficient and optimal response in each patient [124], thereby reducing the recurrence rate of BC. Personalised medicine can be given to patients based on disease conditions and specified biomarkers identified in them. As described in this review, MPs are an ideal biomarker and can be a direct target for drug binding instead of genes that may not reflect the proteins being expressed. Targeting MPs could increase sensitivity, and their localisation makes them accessible to drug binding during therapy. In this review, we propose that MPs are the ideal biomarkers as they could significantly aid the diagnosis of BC. With this in mind, it remains to be clarified whether or not there is indeed a more specific and sensitive membrane protein that is an ideal target for BC treatment. It remains a future endeavour to investigate potential membrane proteins expressed in BC cells that are responsible for tumour development and progression. Further research needs to be conducted to discover additional membrane proteins that could be valuable for targeted therapy in BC and subsequently assist in the development of personalised medicine.

## Figures and Tables

**Table 1:** Hallmarks of cancer and relation with membrane proteins.

No.	Hallmarks of Cancer	Relation with Membrane Proteins
1.	Sustaining proliferative signalling	The cancer cells convey the signals by binding the growth factors to cell-surface receptors (tyrosine kinase domains, a membrane protein).
2.	Evading growth suppressors	In normal cells, the tumour suppressor further suppresses proliferation by coupling cell-surface adhesion molecules (e.g., E-cadherin) to transmembrane receptor tyrosine kinases (e.g., the EGF receptor). Sequestration of growth factor receptors restricts cell division signals. In cancer patients, tumour formation occurs due to the loss of the tumour suppressor.
3.	Resisting cell death	Programmed cell death was resisted by upstream regulators involving the Fas ligand/Fas receptor.
4.	Inducing angiogenesis	Regulators of angiogenesis involve signalling proteins that bind to cell surface receptors.
5.	Activating invasion and metastasis	The expression of the key molecule for cell adhesion, E-cadherin (one type of transmembrane protein), increased and became an antagonist of invasion and metastasis.
6.	Enabling replicative immortality	No membrane proteins were found to be associated, but telomerase, a ribonucleoprotein, was reported to be involved in unlimited proliferation.

Source: [18].

## Data Availability

Not applicable.
